# RAD marker microarrays enable rapid mapping of zebrafish mutations

**DOI:** 10.1186/gb-2007-8-6-r105

**Published:** 2007-06-06

**Authors:** Michael R Miller, Tressa S Atwood, B Frank Eames, Johann K Eberhart, Yi-Lin Yan, John H Postlethwait, Eric A Johnson

**Affiliations:** 1 Institute of Molecular Biology, University of Oregon, 1370 Franklin Blvd., Eugene, Oregon 97403, USA; 2 Institute of Neuroscience, University of Oregon, 1370 Franklin Blvd., Eugene, Oregon 97403, USA; 3 FloraGenex, Inc., 1370 Franklin Blvd., Eugene, Oregon 97403, USA

## Abstract

We constructed a restriction site associated DNA (RAD) marker microarray to facilitate rapid genetic mapping of zebrafish mutations. Using these microarrays with a bulk segregant approach, we localized previously unmapped mutations to genomic regions just a few centiMorgans in length. Furthermore, we developed an approach to assay individual RAD markers in pooled populations and refined one region. The RAD approach is highly effective for genetic mapping in zebrafish and is an attractive option for mapping in other organisms.

## Abstract 2

A RAD marker microarray was constructed to facilitate rapid genetic mapping of zebrafish mutations and used to localize previously unmapped mutations to genomic regions just a few centiMorgans in length.

## Background

Zebrafish (*Danio rerio*) has become an important model system for the study of vertebrate development and human disease, for the assignment of function to genes otherwise known only by sequence, and for the identification of novel functions for genes with previously described functions [1-5]. The success of zebrafish investigation has, in large part, been driven by its amenability to forward genetic approaches [6-8]. Large-scale genetic screens in zebrafish have isolated thousands of mutations that cause an impressive array of phenotypic abnormalities [9,10] (see ZFIN [Zebrafish Model Organism Database] [11] for a full list), and new mutations will continue to be identified in ongoing highly directed genetic screens. The genetic mapping of these mutations is often the critical first step toward elucidating the underlying molecular basis of the biologic process disrupted by mutation. Therefore, developing resources that will accelerate genetic mapping in zebrafish is important [12].

The first eight zebrafish mutations were mapped using a bulk segregant approach with a map constructed from anonymous random amplified polymorphic DNA markers [8]. The same basic approach is commonly used today with microsatellite markers [13,14]. Although these approaches have been effective, they are expensive, labor intensive, and time consuming. We have previously shown that restriction site associated DNA (RAD) marker genotyping is a microarray-based method that allows thousands of polymorphic markers to be screened in parallel [15]. RAD tags are a genome-wide representation of a particular restriction enzyme's recognition sequence by short DNA tags, and DNA sequence polymorphisms that disrupt restriction sites allow RAD tags to serve as high-density genetic markers. The RAD approach has been used to map natural variation in stickleback [15], but it has not yet been applied to map induced mutations in any species.

Here we report the successful application of RAD marker genotyping to mapping of zebrafish mutations. We constructed a zebrafish RAD marker microarray that genotypes thousands of polymorphic markers in parallel. Using a bulk segregant mapping approach, we were able to localize previously unmapped mutations to narrow genomic regions in single hybridizations. In addition, we developed a new polymerase chain reaction (PCR)-based method for genotyping individual RAD markers and rapidly refined the genomic interval for one mutation. From these results, we conclude that the RAD approach is a highly effective, rapid, and inexpensive method for mapping zebrafish mutations and is an attractive option for mapping in other organisms.

## Results and discussion

### Production of the zebrafish RAD marker microarray

**Figure 1:**
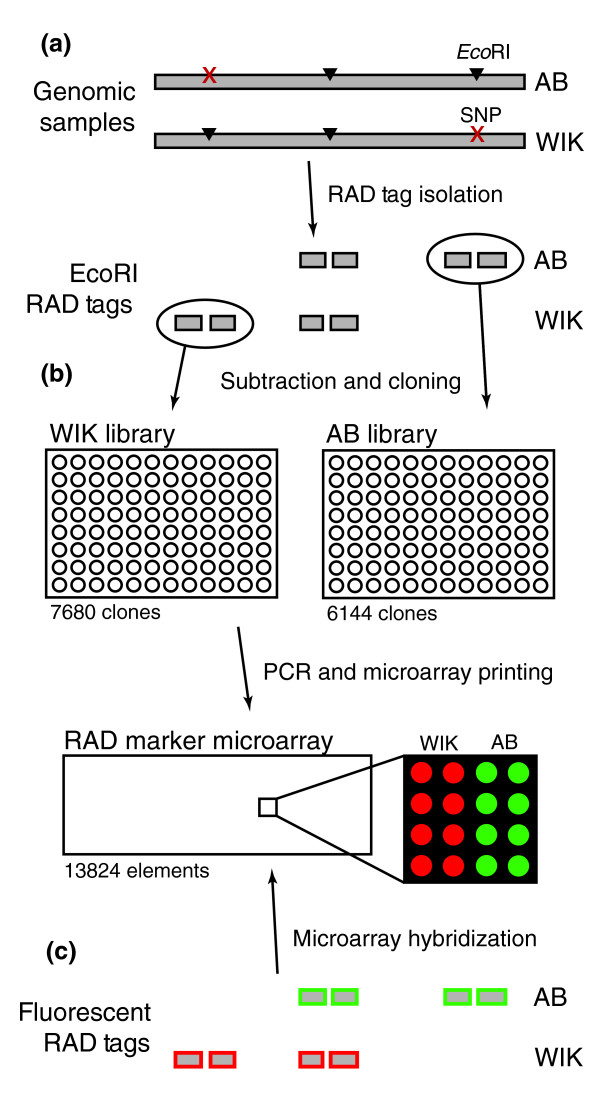
Zebrafish RAD marker microarray construction. **(a) ***Eco*RI restriction site associated DNA (RAD) tag samples were isolated from the AB and WIK strains. **(b) **Clone libraries were generated that were enriched for RAD tags specific to AB or WIK, and these libraries were used to produce a RAD marker microarray. **(c) **AB and WIK RAD tag samples were fluorescently labeled and hybridized directly against each other to the microarray. Elements derived from a WIK-specific RAD tag should have differential hybridization associated with the WIK RAD tag sample. The same should be seen with AB-specific RAD tag elements and the AB RAD tag sample.

One powerful aspect of the RAD approach is that RAD markers can be genotyped on low-cost microarrays that are made from the DNA of two polymorphic RAD tag samples [15]. To construct a zebrafish RAD marker microarray, we first isolated *Eco*RI RAD tag samples from the common laboratory strains AB and WIK [15]. For each strain, genomic DNA was digested with *Eco*RI and biotin linkers were ligated onto the ends. These samples were randomly sheared, leaving only the fragments that were directly flanking a restriction site attached to biotin. The biotin-ended fragments were purified and released from the linkers (Figure 1a). Because RAD tags that are present in both samples will not serve as informative markers, subtractive hybridizations were used to enrich for RAD tags that are specific to either AB or WIK. We modified the previously described subtraction protocol (see Materials and methods, below) [15] with the goal being to produce a microarray with a greater informative marker rate. Random cloning was used to generate libraries from the enriched samples. In all, 7,680 clones were isolated from the enriched WIK sample and 6,144 from the enriched AB sample. PCR was used to amplify these libraries, and the products were spotted on glass slides to yield 13,824 element microarrays (Figure 1b).

To determine the number of AB-by-WIK informative markers typed on this microarray, the AB and WIK RAD tag samples were amplified, fluorescently labeled, and competitively hybridized directly against each other to a microarray (Figure 1c). Using a twofold cut-off identified 3,128 of the 13,824 array elements (22.6%) with strain-specific hybridization, representing a significant increase over the previously described rate of 10% [15]. Also, as expected, array elements for which the increased hybridization was associated with the AB tag sample were isolated specifically from AB, and a similar specificity occurred for the WIK tag sample and the WIK derived array elements (Table 1). Because only a minor modification to the subtraction protocol led to such an increase in the informative marker rate, still more effective subtraction methods should further increase the fraction of informative markers on future RAD marker microarrays.

**Table 1:** Zebrafish RAD marker microarray characterization

	Number of array elements	AB-specific elements	WIK-specific elements	Total polymorphic elements
AB Library	6,144	1,373	14	1,387
WIK Library	7,680	32	1,709	1,741
Both Libraries	13,824	1,405	1,723	3,128 (22.6%)

Restriction site associated DNA (RAD) tags from AB and WIK were fluorescently labeled and competitively hybridized directly against each other to the RAD marker microarray. Elements with a differential hybridization greater than twofold were counted as polymorphic.

Although the production method we used will result in an array that is optimized for AB-by-WIK mapping crosses, the phylogenetic relationship between common zebrafish strains [16] suggests that a large number of informative markers could also be typed in other strains. To test how effective the current RAD array is for other genotypes, we isolated a RAD tag sample from the common strain TU and compared it with the AB and WIK samples. A direct hybridization of TU and AB revealed differential hybridization of 1,815 elements, whereas a direct hybridization of TU and WIK revealed differential hybridization of 1,928 elements. These results are consistent with the previously described phylogenetic relationships of these strains [16]. Furthermore, although the array is optimized for AB-by-WIK mapping crosses, the number of informative markers available between any pair-wise combination of AB, TU, and WIK should be sufficient for bulk segregant mapping. Therefore, the current RAD array should have wide applicability for mapping crosses between a variety of zebrafish strains.

### Bulk segregant mapping

**Figure 2:**
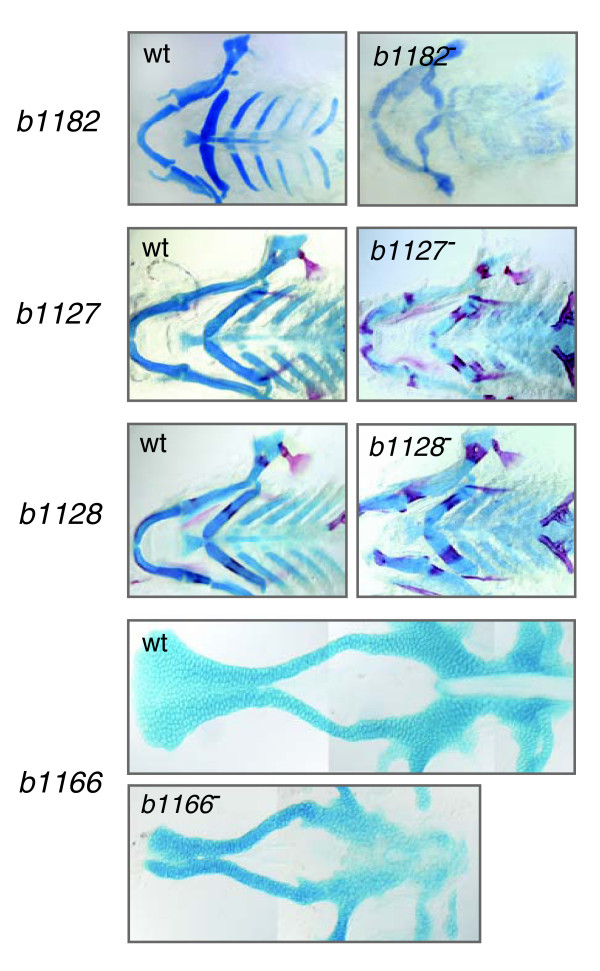
Phenotypes of the four recessive mutations that were mapped. The *b1127 *and *b1128 *mutations cause premature ossification of craniofacial chondral bones and were isolated in the TU background. The *b1182 *mutation diminishes the differentiation of cartilage and bone in specific elements of the neurocranium and pharyngeal arches, and was isolated in TU. The *b1166 *mutation causes a shortened body axis, abnormal somite morphology, and clefting of the anterior neurocranium, and was isolated in AB. Cartilage is stained with Alcian blue, and bone with Alizarin red. wt, wild type.

We explored the utility of the array for bulk segregant mapping by attempting to localize four previously unmapped recessive mutations that we had recovered from the ongoing ENU-mediated forward genetic screen at the University of Oregon [17-19] (Figure 2). The *b1127 *and *b1128 *mutations cause premature ossification of craniofacial chondral bones and were isolated in the TU background and out-crossed to AB for mapping. The *b1182 *mutation, also isolated in a TU background, diminishes the differentiation of cartilage and bone in specific elements of the neurocranium and pharyngeal arches, and was out-crossed to WIK for mapping. The *b1166 *mutation, causing a shortened body axis, abnormal somite morphology, and clefting of the anterior neurocranium, was isolated in AB and out-crossed to WIK for mapping.

**Figure 3:**
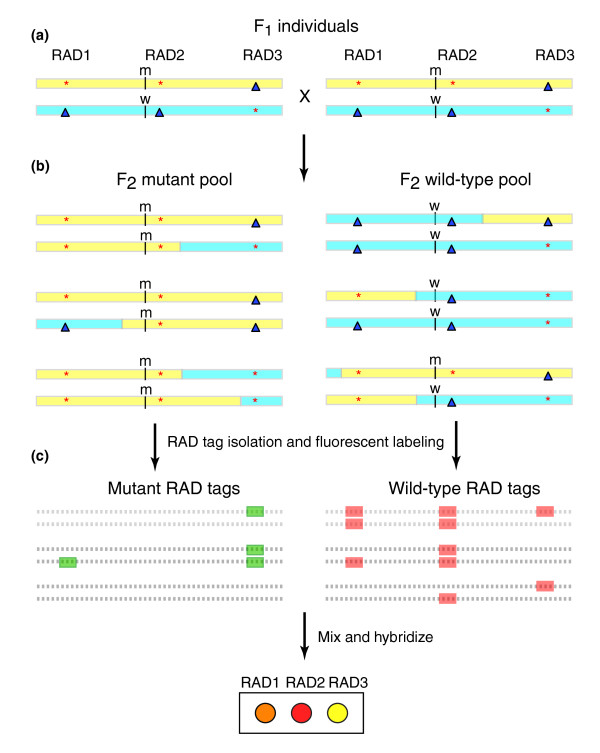
RAD mapping using bulk segregant analysis. **(a) **F_1 _fish heterozygous for the mutation and a polymorphic mapping strain are crossed together. **(b) **The F_2_, a few of which are shown here, will contain a large variety of recombinant chromosomes. Pools of DNAs from either mutant or phenotypically wild-type animals are made. Marker RAD2, for example, is closely linked to the wild-type allele of the mutant locus, and therefore (except for rare recombinants) will appear in the wild-type pool but not in the mutant pool. **(c) **Restriction site associated DNA (RAD) tags are isolated from each of the pools, fluorescently labeled, and hybridized to the RAD marker microarray. Markers unlinked to the mutant locus should be in approximately equal quantities in both pools. As markers become closer to the mutation, the fraction of individuals that have one or the other RAD marker allele will increase, resulting in array elements with high or low ratios of red to green fluorescence.

For mapping, we crossed fish heterozygous for a mutation to individuals of a different strain to obtain F_1 _individuals, and mated F_1 _by F_1 _to obtain diploid F_2 _embryos (Figure 3a). For each mapping cross, we extracted DNA from individual F_2 _embryos that were either phenotypically mutant or phenotypically wild-type. DNA aliquots from 20 to 30 mutant embryos were pooled together and DNAs from 20 to 30 wild-type embryos were pooled (Figure 3b). From each pool, RAD tag samples were isolated, fluorescently labeled, and competitively hybridized directly against each other to the RAD microarray (Figure 3c). One hybridization experiment was performed for each mutation. Closely linked RAD tags that were only present in the wild-type parent will be absent in the mutant pool and present in heterozygous or homozygous states in the wild-type pool, and show up as high-ratio red array elements. Closely linked RAD tags that were only present in the mutant parent will be completely present in the mutant pool and also present in heterozygous individuals in the wild-type pool, and show up as array elements with a green fluorescence threefold higher than red fluorescence. Markers that segregate independently of the mutation will appear yellow because they will be present in equal quantities in both mutant and wild-type pools. In principle, the relative hybridization ratio will be a function of the distance between the marker and mutation.

To identify the location of the mutation, we sequenced five to ten array elements that appeared to be strongly associated with each mutant phenotype (see Materials and methods, below). Although RAD markers from either parent can be used to identify the region closely linked to the mutation [15], we sequenced only WIK markers because they produce higher ratios and they allow for RAD Amp individual marker analysis (see below). The RAD microarray data from one of the mutants is shown in Table 2. A blastn search against the Zv6 assembly of the zebrafish genome [20] identified the genomic assembly positions of the linked markers, which in turn allowed determination of the approximate genetic map position. We confirmed the results by genotyping individual F_2 _embryos with microsatellite markers located in or near the regions specified by the RAD mapping.

**Table 2:** *b1166 *RAD microarray mapping data

Marker name	Experiment A: *b1166 *(wild type)/*b1166 *(mutant)	Experiment B: WIK/AB	Experiment A/experiment B	Zv6 location	Completely associated (RAD Amp)
WIK18I13	5.78	8.91	0.65	Chr:25 (23.7 Mb)	No
WIK13P08	5.12	5.35	0.96	Chr:25 (12.0 Mb)	No
WIK20J21	4.97	2.78	1.78	Chr:25 (24.9 Mb)	Yes
WIK08N22	4.57	3.60	1.27	Chr:9 (30.9 Mb)	n.t.
WIK05E18	4.21	4.87	0.86	Chr:25 (16.6 Mb)	No
WIK18C01	3.71	3.73	0.99	Chr:25 (18.6 Mb)	Yes
WIK11I12	3.57	3.09	1.16	Chr:25 (18.2 Mb)	Yes
WIK07C02	3.56	3.51	1.01	Chr:25 (22.7 Mb)	No
WIK07B09	3.46	3.22	1.08	Chr:21 (50.0 Mb)	n.t.
WIK02K07	3.06	4.26	0.72	Chr:1 (17.9 Mb)	n.t.

Normalized ratios are shown for microarray experiments. Experiment A is the hybridization of *b1166 *wild-type pool restriction site associated DNA (RAD) tags verses *b1166 *mutant pool RAD tags. Experiment B is the hybridization of AB RAD tags verses WIK RAD tags. The marker locations according to Zv6 assembly of the zebrafish genome are shown. In the 'Completely associated (RAD Amp)' column, entries are as follows: 'Yes' for RAD markers for which there was no wild-type allele in the *b1166 *mutant pool according to RAD Amp; 'No' for RAD markers for which there was wild-type allele present in the *b1166 *mutant pool according to RAD Amp; and 'n.t.' for RAD markers that were not tested using RAD Amp. RAD markers that are completely associated with the mutation often have higher ratios of experiment A to experiment B.

**Figure 4:**
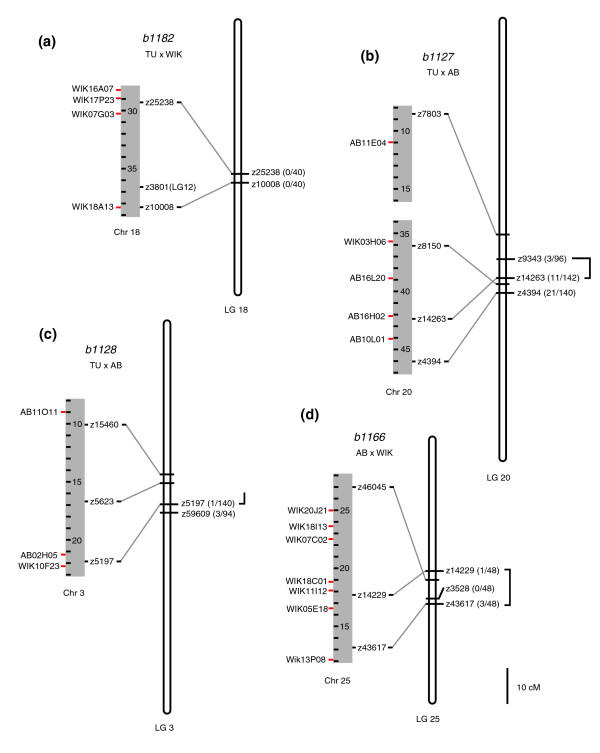
Genomic locations of RAD markers linked to zebrafish mutations. Wide gray vertical bars represent chromosome (Chr) regions based on the Ensembl Zv6 genome assembly (megabase positions are shown); restriction site associated DNA (RAD) marker positions (red tick marks) are indicated to the left, and microsatellite markers to the right. Narrow, vertical bars represent linkage groups (LGs) based on the MGH mapping panel [27]. Lines connect the genome assembly and genetic map positions of microsatellite markers. Crossed lines indicate inconsistencies between the genome assembly and genetic map. Microsatellite markers were used to confirm the RAD mapping results. The recombination patterns in individual embryos identified regions containing the mutations (black brackets). Microsatellite markers are shown to the right of the linkage group. The number of recombination events between the marker and mutation over the total number of meioses is shown in parentheses. **(a) ***b1182 *was isolated in the TU background and out-crossed to WIK for mapping. RAD marker positions suggest that *b1182 *is between 42.1 cM (z25238) and 44.2 cM (z10008) on LG 18. Recombination patterns in individual embryos confirm that *b1182 *is within or very near this region. **(b) ***b1127 *was isolated in the TU background and out-crossed to AB. RAD marker positions suggest that *b1127 *is between 59.2 cM (z7803) and 75.7 cM (z4394) on LG 20. Individual genotypes confirm *b1127 *is within this region, between z9343 (66.2 cM) and z4304 (72.2 cM). **(c) ***b1128 *was isolated in the TU background and out-crossed to AB. RAD marker positions suggest that *b1128 *is between 42.0 cM (z15460) and 50.4 cM (z5197) on LG 3. Individual genotypes confirm *b1128 *is within this region, approximately 0.7 cM from z5197 (50.4 cM). **(d) ***b1166 *was isolated in the AB background and out-crossed to WIK. RAD marker positions suggest that *b1166 *is between between 36.4 cM (z14229) and 45.7 cM (z43617) on LG 25. Individual genotyping confirms that *b1166 *is within this region.

For *b1182 *we sequenced five RAD markers that appeared to be linked to the mutation. Four sequences were located on chromosome 18 in the region that corresponds to 42.1 to 44.2 cM on linkage group (LG) 18 of the Boston MGH Cross (MGH) meiotic map [13] (Figure 4a). The fifth marker was located on chromosome 20 in Zv6. Recombination patterns of individual embryos scored for microsatellite markers z25238 and z10008 confirmed that *b1182 *is within or very near the region between 42.1 and 44.2 cM on LG 18.

For *b1127 *we sequenced seven RAD markers. Five of the seven were located in the region of chromosome 20 that corresponds to the interval from 59.2 to 75.7 cM on LG 20 (Figure 4b). Another marker had no significant blastn hit against Zv6, and the seventh marker was located on chromosome 1 in Zv6. Genotyping individual F_2 _embryos confirmed that *b1127 *is within the region between z9343 (66.2 cM) and z4304 (72.2 cM) on LG 20.

For *b1128 *we sequenced five RAD markers. Three markers were located in the region of chromosome 3 that corresponds to between 42.0 and 50.4 cM on LG 3 (Figure 3c). The other markers had no significant blastn hits against Zv6. Individual genotypes confirmed that *b1128 *is within this region, approximately 0.7 cM from z5197 (50.4 cM).

For *b1166 *we sequenced ten RAD markers, and seven were located in the region of chromosome 25 that corresponds to between 36.4 and 45.7 cM on LG 25 (Figure 3d). The three others were located on different chromosomes in Zv6. Individual genotypes confirmed that *b1166 *is within the region between z14229 (36.4 cM) and z43617 (45.7 cM) on LG 25.

Taken together, these findings demonstrate the ability of RAD microarrays to localize previously unmapped mutations rapidly and accurately. For the mutations we mapped, the average region size identified was 9.1 cM, or 0.4% of the zebrafish gene map [13], and microsatellite markers confirmed that the mutations were within these regions. As expected, the RAD marker density was greatest with the AB-by-WIK cross (*b1166*). However, an equivalent mapping resolution was achieved with crosses involving TU, thus confirming the applicability of RAD arrays for mapping with strains not used in the array production process.

### RAD Amp: a PCR-based method for genotyping individual RAD markers

Although the RAD microarray is able to localize mutations to narrow genomic regions rapidly, subsequent fine mapping is needed to identify the causative loci. Unlike the initial stages of mapping, fine mapping requires typing a small number of closely linked markers in a large number of individuals. Large numbers of microsatellite markers are available to refine the regions identified by microarray [13]. In practice, however, a significant fraction of available microsatellites are not polymorphic in a given cross, and some genomic regions contain only a low density of markers. In many cases it would be convenient to use RAD markers directly to refine the regions identified by microarray. To facilitate this, we developed a PCR-based method for genotyping individual RAD markers. This method allows for the detection of rare wild-type alleles in a pooled mutant population.

**Figure 5:**
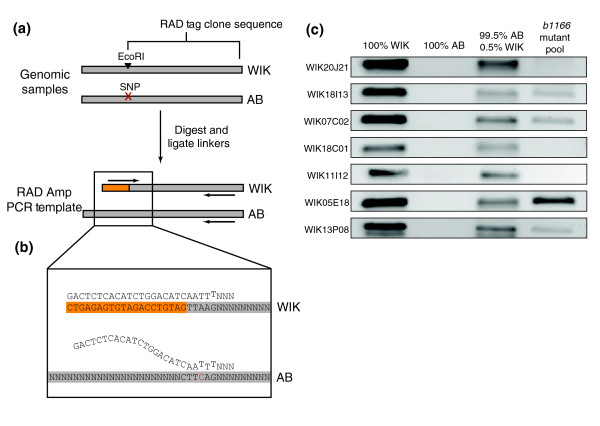
RAD Amp: a PCR-based method for genotyping individual RAD markers. **(a) **Genomic DNA is digested, ligated to short linkers (orange bar), and used as polymerase chain reaction (PCR) template for restriction site associated DNA (RAD) Amp. **(b) **RAD Amp primer design takes advantage of primer-template mismatching to discourage aberrant extension in the absence of linker. In the AB sample, the polymorphism disrupting the restriction site is red. **(c) **RAD Amp was performed using primers specific to the seven RAD markers linked to *b1166*. Amplification using these primers with WIK and AB template showed WIK-specific allele detection. RAD Amp detected WIK alleles in a pool containing 0.5% WIK and 99.5% AB DNA. WIK alleles, from the wild-type parent, were detected in a pool of 24 F_2 _mutant individuals from the *b1166 *mapping cross at WIK18I13, WIK07C02, WIK05E18, and WIK13P08. No WIK alleles were detected at WIK20J21, WIK18C01, or WIK11I12, suggesting that these markers are the more closely linked to the mutation.

For this method, genomic DNA is digested with a particular restriction enzyme and linkers are ligated to the fragment ends. The sequence of specific polymorphic RAD tag clones is used to design PCR primers such that one primer overlaps the restriction site and contains sequence from both genomic and linker DNA, whereas the other primer is a few hundred base pairs away (Figure 5a). The digested and ligated sample is used as a PCR template with these primers. Because one primer binds to both linker and genomic DNA, efficient binding and extension will only take place if a restriction site is present at that position to allow ligation of the linker. Furthermore, the primer that overlaps the linker is designed with a mismatch near the 3' end to discourage aberrant extension in absence of linker (Figure 5b).

We tested this method on the seven RAD markers we identified as closely linked to *b1166 *(Figure 4d). These markers have increased hybridization associated with WIK RAD tags relative to AB tags, and should therefore represent instances in which a restriction site is present in WIK and linked to the wild-type allele but absent from the mutant background AB (Figure 5a). Thus, applying RAD Amp to each of the seven markers should result in amplification from WIK samples but not AB. To test this prediction, AB and WIK genomic samples were digested and linkers were ligated onto the ends of digestion fragments. These samples were used as PCR template for amplification with primers designed from sequences of each of the seven *b1166*-linked RAD markers. As expected, a PCR product was generated for each marker using WIK as template but not AB (Figure 5c, lanes 1 and 2). We next tested whether RAD Amp could be used to detect rare copies of the WIK allele (having arisen from a rare recombination event) in a pool of predominately AB alleles. We made a genomic DNA mix containing 99.5% AB and 0.5% WIK genotypes, simulating one heterozygote in a population of 100 diploid individuals. Following digestion and ligation, this genomic mixture was used as PCR template with the RAD Amp primers tested above. For each marker, the method successfully detected the rare WIK allele (Figure 5c, lane 3), thus confirming the ability of RAD Amp to detect rare recombinant alleles in a pooled sample of segregants.

Because *b1166 *was generated in the AB background, mutant individuals will rarely contain WIK alleles at the markers most closely linked to that mutation. To refine quickly the genomic region that was previously identified as being linked to the mutation, we used the RAD Amp approach to determine whether WIK alleles were present in a pool of 24 mutants at the seven *b1166*-linked RAD markers. As described above, the pooled DNA was digested and linkers were ligated to the fragment ends, and this sample was used as PCR template. PCR product was generated for four of the markers (WIK18I13, WIK07C02, WIK05E18, and WIK13P08), demonstrating WIK allele presence in the mutant pool at these loci. However, no product was generated for the remaining three markers (WIK20J21, WIK18C01, and WIK11I12), demonstrating the pool contained no WIK allele at these loci (Figure 5c and Table 2). These results suggest that the three markers with no amplification product (WIK20J21, WIK18C01, and WIK11I12) are more closely linked to *b1166 *than the others. Although the genomic assembly does not place WIK20J21 next to WIK18C01 and WIK11I12, the genetic map is inverted with respect to the assembly (Figure 4d), suggesting that the assembly marker order is incorrect. Thus, the RAD Amp approach allowed us to refine rapidly the genomic region identified by microarray. Enlarging the size of the pool or testing similarly sized pools from different individual segregants should further narrow the region.

These results show that the RAD Amp approach can reliably detect the presence of rare alleles in a large population of individuals, allowing rapid screening of pooled populations. The bulk segregant array hybridization identifies tightly linked RAD markers, and subsequent RAD Amp experiments rapidly screen those markers in the same and/or additional pools to identify markers with the tightest association to the mutation. The RAD Amp approach may be a good alternative to using existing microsatellites to refine the regions identified by microarray. As the density of linked markers increases, it becomes advantageous to examine large populations for informative breakpoints. Individual genotyping of markers in a large population is expensive and time consuming, whereas the identification of completely linked RAD markers in a pooled population using RAD Amp can be carried out rapidly. Furthermore, the RAD Amp approach will be very useful for organisms in which a large set of individually typed markers is not available. Therefore, RAD Amp is a rapid and useful additional step in mapping with RAD markers.

### Future improvements

A number of improvements can increase the power of the RAD approach. For one, we found many microsatellite alignment inconsistencies between the genomic assembly and the MGH meiotic map [13]. The high degree of similarity between independently generated meiotic maps suggests that errors in the genetic map are infrequent [21]. Thus, these alignment inconsistencies are probably due to assembly errors. Assembly improvements would better define the regions identified in mapping experiments. Also, we plan to sequence all elements in the microarray. This not only would eliminate a step in our current process, but in addition it would allow us to plot hybridization ratios along a chromosome. The peak of the hybridization ratio would identify the region with the greatest association, and hence the most likely mutant locus.

Based on genome size [22] and average single nucleotide polymorphism frequency [16], more than 20,000 informative RAD markers are expected to be available for a restriction enzyme with a six nucleotide palindromic recognition sequence [15]. RAD marker microarrays could be produced that contain more informative markers, utilize a different restriction enzyme, or are optimized for different strains. Even if producing a microarray with a larger number of informative markers did not improve microarray mapping resolution, it would provide an increased marker density for the RAD Amp technique, and thus increase the achievable mapping resolution. The clone and printing libraries needed for a RAD marker microarray can be generated in a few weeks, with the greatest expense being the plastic ware, and the printing plates contain enough material to print thousands of microarrays. Alternatively, high-density genomic tiling microarrays could be used for typing RAD markers as they become more widely available. Whole genome tiling microarrays would probably type the majority of RAD markers for an enzyme, could be used for a variety of enzymes, and would not be strain specific. Therefore, these arrays would be a powerful yet more costly platform for RAD marker genotyping.

Many technical aspects of the RAD methodology could be improved. We used ligation-mediated PCR to amplify RAD tags before fluorescent labeling and hybridization. Improved amplification strategies that reduce noise in chromatin immunoprecipitation microarray (ChIP-chip) experiments [23,24] may also improve RAD mapping experiments. Alternatively, increasing the genomic starting material would allow increased isolation of RAD tag DNA such that RAD tags could be directly labeled without amplification.

## Conclusion

In this report we describe the production of a zebrafish RAD marker microarray and show that this microarray can facilitate rapid and inexpensive genetic mapping of zebrafish mutations. We demonstrated this by rapidly localizing four previously unmapped mutations to narrow genomic intervals. Furthermore, we developed a PCR-based approach for genotyping individual RAD markers and used it to refine one region rapidly. The utility of the RAD Amp approach is derived from its ability to detect rare alleles in a pooled population. Thus, RAD microarrays allow the screening of thousands of markers in a single hybridization, and the RAD Amp approach allows the screening of a large number of individuals in a single PCR reaction.

Here we applied the RAD approach to zebrafish, which is a well developed model organism. The ease of RAD microarray construction and genotyping makes the RAD approach an attractive option for mapping in other model organisms. Furthermore, the ability to develop RAD microarrays inexpensively and without prior sequence information or other costly resources makes the RAD approach an attractive option for high-resolution genotyping in nonmodel organisms, because the identification of a large number of linked high-density markers simplifies screening large-size genomic fragment panels or the creation of genetic maps using the RAD markers.

## Materials and methods

### Zebrafish microarray production

AB and WIK genomic samples were each prepared by combining genomic DNA from one male and one female. Characteristics of the wild-type strains used in these experiments are available at ZFIN [25]. These samples were used for the enriched RAD marker microarray production process. Briefly, *Eco*RI RAD tag samples were isolated from these genomic samples, and subtractive hybridizations between the RAD tag samples were performed to enrich for informative RAD tags. Clone libraries were generated from these enriched RAD tag samples. These clone libraries were used as templates for PCR, the products of which were spotted to create the microarray. Previously described methods [26] were used for this process, with the following modifications. First, in the subtractive hybridizations, 250 ng of tester, 5 μg of driver, and no glycogen were used; also, three rounds of subtraction were performed. Second, the microarray prehybridization solution was 5 × saline sodium citrate, 0.1% sodium dodecyl sulfate, and 0.1 mg/ml bovine serum albumin.

### RAD tag isolation, labeling, and hybridization

RAD tag isolations, labeling, and hybridization were performed as previously described [26]. Briefly, genomic DNA was digested and biotin linkers were ligated onto the ends of the digested fragments. These samples were randomly sheared via sonication to an average size of about 500 base pairs, leaving only the fragments that were directly flanking a restriction site attached to the linkers. The biotin-ended fragments were purified using streptavidin beads and released from the linkers. Following amplification by ligation-mediated PCR, two RAD tag samples were fluorescently labeled and hybridized directly against each other to a microarray. Following hybridization, the arrays were scanned to generate images of the fluorescent signal intensities. For the mapping experiments, the mutant pool and wild-type pool RAD tag samples were fluorescently labeled with Alexa Fluor^® ^555 and Alexa Fluor^® ^647 (Invitrogen, Carlsbad, CA, USA) respectively.

### Mapping analysis

F_1 _mapping heterozygotes were generated by outcrossing mutant carriers. These mapping heterozygotes were in-crossed to produce the diploid F_2 _embryos used for mapping. The four mutations we mapped were recessive in diploid embryos. For this reason, the informative RAD tags that are highly linked to the dominant wild-type allele should rarely be present in mutant embryos, but they should nearly always be present in either the heterozygous or homozygous state in wild-type embryos. In contrast, tags that are highly linked to the mutant allele should nearly always be in the homozygous state in mutant embryos, but they should also be present in the heterozygous state in two-thirds of the phenotypically wild-type embryos. Therefore, tags that are linked to the wild-type allele should produce larger hybridization differences than tags linked to the mutant allele. For this reason, we limited our analysis to the wild-type specific RAD tags. Wild-type specific elements with hybridization differences greater than about threefold were chosen. This threshold provided approximately five to ten elements per mutation. Plasmids isolated from the corresponding clones were insert sequenced and a blastn search was used to locate the sequences in the Ensembl Zv6 genome assembly [22]. For each mutation, the majority of the sequences clustered in a narrow region of the genome assembly. We performed a Monte Carlo simulation of marker clustering and determined that the probability of finding three markers within a 10 megabase region by chance alone to be less than 1 in 10,000 for five markers sequenced, or less than 1 in 1,000 when ten markers are sequenced. The Ensembl genome browser was used to identify microsatellite markers located in the region. The genotyping of putatively linked microsatellites in the mapping cross confirmed the linkage and identified the corresponding region of the MGH genetic map [27].

### RAD Amp

Genomic DNA samples were digested with *Eco*RI (New England Biolabs, Ipswich, MA, USA) and short linkers (5'-GACTCCTCGACTCTCACATCTGGACATA-3', 5'-Phos-AATTTATGTCCAGATGTGAGAGTC-3') were ligated to the fragment ends using previously described conditions [26]. PCR reactions were carried out in 50 μl under the following conditions: 50 ng template, 1 × Thermopol Buffer (New England Biolabs), 2.5 U Taq (New England Biolabs), 0.2 mmol/l dNTPs (Fermentas, Burlington, Ontario, Canada), 0.4 μmol/l forward primer, and 0.2 μmol/l reverse primer. Touchdown amplification was applied, with primer annealing temperature dropping 2°C every other cycle from 65°C to 55°C, after which 25 additional cycles were carried out [28]. A volume of 5 μl of amplified sample was run on a 2% agarose gel. Primers were designed with the following scheme. Forward primers contained 20 nucleotides complementary to the linker sequence and eight nucleotides complementary to the genomic sequence. A mismatch was introduced near the 3' end to discourage aberrant extension in the absence of linker [29]. Reverse primers were designed from RAD tag clone sequence to produce products about 300 base pairs in length (Figure 5b).
